# Lifestyle patterns associated with diet, physical activity, body mass index and amount of recent weight loss in a sample of successful weight losers

**DOI:** 10.1186/1479-5868-9-79

**Published:** 2012-06-26

**Authors:** Paul T Fuglestad, Robert W Jeffery, Nancy E Sherwood

**Affiliations:** 1 Division of Epidemiology and Community Health, School of Public Health, University of Minnesota, 1300 S 2nd Street, suite 300, Minneapolis, MN 55454, USA; 2 HealthPartners Research Foundation, 8170 33rd Ave. S., Mail stop 21111R, Bloomington, MN 55425, USA

**Keywords:** Lifestyle patterns, Body mass index, Weight loss, Diet, Physical activity

## Abstract

**Background:**

Research suggests that the interaction between biological susceptibility and environmental risk is complex and that further study of behavioral typologies related to obesity and associated behaviors is important to further elucidate the nature of obesity risk and how to approach it for intervention. The current investigation aims to identify phenotypical lifestyle patterns that might begin to unify our understanding of obesity and obesity related behaviors.

**Methods:**

Individuals who had recently lost substantial weight of their own initiative completed measures of intentional weight control behaviors and lifestyle behaviors associated with eating. These behaviors were factor analyzed and the resulting factors were examined in relation to BMI, recent weight loss, diet, and physical activity.

**Results:**

Four meaningful lifestyle and weight control behavioral factors were identified— regularity of meals, TV related viewing and eating, intentional strategies for weight control, and eating away from home. Greater meal regularity was associated with greater recent weight loss and greater fruit and vegetable intake. Greater TV related viewing and eating was associated with greater BMI and greater fat and sugar intake. More eating away from home was related to greater fat and sugar intake, lower fruit and vegetable intake, and less physical activity. Greater use of weight control strategies was most consistently related to better weight, diet, and physical activity outcomes.

**Conclusions:**

Compared to the individual behavior variables, the identified lifestyle patterns appeared to be more reliably related to diet, physical activity, and weight (both BMI and recent weight loss). These findings add to the growing body of literature identifying behavioral patterns related to obesity and the overall weight control strategy of eating less and exercising more. In future research it will be important to replicate these behavioral factors (over time and in other samples) and to examine how changes in these factors relate to weight loss and weight maintenance over time.

## Background

The high profile of obesity as a health issue continues to kindle interest in better understanding the factors that are responsible for obesity susceptibility and difficulty in adhering to lifestyle treatment. Long before the current epidemic was recognized it was clearly established that obesity runs in families and, therefore, that underlying biology is an important factor [1-3]. The unfolding population epidemic, likewise, has underscored the importance of factors in the environment that have recently resulted in dramatic changes in risk over relatively short periods of time [4,5]. As research progresses, however, it is becoming clear that the interaction between biological susceptibility and environmental risk is not a simple one and that further study of behavioral typologies of phenotypes predicting obesity risk may be important to further elucidate the nature of risk and how to approach it for intervention [6-8]. An ever increasing list of variables at the interface of biology and environment is now being reported, mostly from cross-sectional and longitudinal studies of populations with different characteristics, e.g., general population, weight-loss study volunteers, and successful losers, and with different age and ethnic characteristics. Among the variables most consistently associated with obesity risk are lifestyle issues like where and what people eat (e.g., fast food, sugar-sweetened beverages, watching TV), when they eat (e.g., meal skipping), and intentional weight management practices (e.g., self-weighing and meal planning) [9-19].

Although studies have been able to identify various practices associated with obesity risk, there is great heterogeneity in results with even relatively consistent behaviors such as intake of fast food [17]. Furthermore, many studies tend to focus on a limited number of behaviors, and even those with a broader coverage of behaviors have seldom considered the interrelations among behavioral practices. One reason for the heterogeneity in results may be that by focusing on single behavioral indicators, researchers miss the integrated general behavioral patterns that are associated with weight, diet, and physical activity.

Building on prior research suggesting that health behaviors tend to be interrelated [20-24], the current paper presents an analysis of the relationships among a diverse array of variables related to obesity and associated behaviors (i.e., diet and physical activity) with the aim of beginning to identify phenotypical lifestyle patterns that might unify our understanding of obesity, diet, and physical activity. The population under consideration is a unique one, being comprised of adults who have recently lost significant weight on their own initiative and have volunteered for a study intended to help them keep it off. The potentially phenotypical variables fall into the following broad behavioral classes: lifestyle behaviors associated with eating (e.g., eating at fast food restaurants and convenience stores, frequency of meals and snacks, and TV viewing) and intentional weight control behaviors (e.g., counting calories, planning meals and exercise to manage weight). Recent research by Sciamanna et al. [18] underscores the importance of considering the unique relations of behavioral practices to multiple aspects of obesity and weight change (e.g., weight loss and weight maintenance). In this respect, the present analysis examines the outcome variables of weight, defined by BMI at study entry, the amount of weight lost in the recent weight loss episode that qualified participants for study eligibility, and behaviors most proximal to weight regulation, namely diet and physical activity. Utilizing factor analysis, we aim to identify unifying themes of behavior and self-regulation that relate to obesity, success in self-initiated weight loss, diet, and physical activity.

## Methods

### Participants

Participants were recruited for this study from the general population through public advertisement in the St. Paul/Minneapolis area in the USA. The key criterion for study entry was having intentionally lost at least 10% of body weight in the past year. The total sample included 419 adults (82% female; 87% non-Hispanic white; 64% college or graduate degree; age *M* = 47; BMI *M* = 28; % weight loss *M* = 16). Consent was obtained from all participants and the University of Minnesota’s Institutional Review Board Human Subjects Committee approved all protocols. For additional details of participant characteristics, recruitment, and intervention procedures see Sherwood et al. [25].

### Measures

Table 1 shows descriptive statistics for all measures. Variables in the two lifestyle categories as well as the outcome variables are defined below.

**Table 1:** Baseline descriptive statistics for study variables

***Lifestyle Behaviors***	
Eat breakfast (daily)	62%
Eat lunch (daily)	62%
Eat dinner (daily)	72%
Eat after 7 p.m. (3 +/week)	60%
Eat snacks with TV (3 +/week)	37%
Eat meals with TV (3 +/week)	46%
TV viewing (2 + hr/day)	47%
Eat food from work (1 +/week)	43%
Eat food from sit down (1 +/week)	70%
Eat fast food (1 +/week)	48%
Eat food from convenience store (1 +/week)	15%
Eat food from fundraiser (1 +/week)	1%
*Weight control Behaviors*	
Self-weighing (daily +)	47%
Write down calorie content (sometimes +)	25%
Write down exercise (sometimes +)	40%
Use meal replacements (sometimes +)	21%
Plan meals (often +)	66%
Plan exercise (often +)	62%
*Outcome Variables*	
Body mass index	28.5 (4.9)
% weight lost at baseline	16.2 (5.3)
Fruits (servings per day; excluding juice and fruits in foods such as pies)	1.6 (1.2)
Vegetables (servings per day; excluding starchy vegetables and vegetables in foods such as stews or pot pies)	1.1 (.85)
Discretionary fat (grams per day)	44.9 (27.9)
Added sugars (teaspoons per day)	11.4 (9.0)
Physical Activity (kcal per week)	1998 (1688)

Percentages of participants performing a given behavior are shown for lifestyle and weight control behaviors. Means and standard deviations are shown for continuous outcome variables.

#### Lifestyle behaviors

Participants indicated how many times during the past week they 1) ate breakfast, 2) ate lunch, 3) ate dinner, 4) ate after 7 p.m., 5) ate a snack while watching TV, 6) ate a meal while watching TV, 7) ate food at work (provided by an employer or other employee), 8) ate food prepared at a fast food restaurant, 9) ate food prepared at a sit-down restaurant, 10) purchased food at a convenience store/gas station, and 11) purchased food for a fundraiser. Response options for each question were *0*, *1 or 2*, *3 or 4*, *5 or 6*, and *7+*. Additionally, participants indicated how many hours of TV they watch on the average weekday and weekend day using the response options *0*, *<1*, *1*, *2*, *3*, *4*, and *5+*. After appropriate weighting, weekday and weekend viewing were combined to form an index of average daily viewing.

#### Weight control strategies

Participants reported how often they weigh themselves using the response options *never*, *once a year or less*, *every couple of months*, *every month*, *every week*, *every day*, and *more than once a day*. They also reported how often they 1) write down the calorie content of the foods they eat, 2) write down the amount and type of exercise they do, 3) use meal-replacement products to manage their weight, 4) plan their meals to manage their weight, and 5) plan their exercise to manage their weight using the response options: *never*, *rarely*, *sometimes*, *often*, and *very often*.

#### BMI

Weight and height were measured in person with participants in light clothing without shoes (Seca 770 Medical Scale; Seca 214 Portable Height Rod). BMI (kg/m^2^) was computed.

#### Weight loss

During the initial phone screening, weight loss in the last year was computed by subtracting self-reported current body weight from self-reported highest body weight during the past year. Weight loss was also computed at baseline by subtracting measured baseline body weight from self-reported highest body weight. The later variable is used in subsequent analysis as the measure of recent weight loss. Adopting procedures from the National Weight Control Registry (NWCR) [26], potential participants were required to document their recent loss (e.g., “before-and-after” photographs, names of individuals able to verify weight loss). This was done to increase assurance of the veracity of their self-reported weight loss. Amount of recent weight loss was unrelated to BMI (*r* = -.07, *p* = .13).

#### Diet

Dietary intake was assessed using the National Cancer Institute's Web-based Diet History Questionnaire (DHQ). Several studies have documented the reliability and validity of the paper-and-pencil version of this measure [27,28], and one advantage of the web-based DHQ is that respondents cannot complete the questionnaire with missing or inconsistent responses. The DHQ asks about the frequency of eating and drinking 124 items over the past year and includes portion size and dietary supplement questions. The data is then analyzed using software developed by NCI to yield nutrient intake and food group servings. Servings per day of fruits (excluding juice and fruits in other foods such as pies) and vegetables (excluding starchy vegetables and vegetables in other foods such as stews or pot pies) were combined (average of z-scores) to form an index of more healthful eating, whereas intake of discretionary fat (grams per day) and added sugars (teaspoons per day) were combined (average of z-scores) to form an index of less healthful eating.

#### Physical activity

Physical activity was assessed using the Paffenbarger Physical Activity Questionnaire [29]. This instrument asks individuals to indicate the number of city blocks walked, flights of stairs climbed, and light (5 kcal/min), medium (7.5 kcal/min), and heavy (10 kcal/min) leisure time activities in the past week. The caloric expenditure from each of these activities was summed to estimate total kcal of energy expenditure per week (beyond basal metabolic rate). The Paffenbarger questionnaire has been shown to have satisfactory reliability and predictive validity [30,31].

### Analysis plan

The main goal of the present research was to examine the relation of lifestyle patterns to diet, physical activity, BMI, and weight loss in the past year. Multiple regression was used when lifestyle variables were examined continuously, and ANOVA was used when lifestyle variables were examined categorically. Initial analysis examined the associations of individual behaviors with outcomes of interest. Factor analysis was then used to identify general lifestyle patterns. Next, the associations of these lifestyle factors with BMI, recent weight loss, diet, and physical activity were examined. All analyses were performed using SPSS. Covariates included in the analyses were sex, age, marital status (married versus not), and race (non-Hispanic white versus not). Cohen’s *d* is reported as an effect size indicator for mean comparisons.

## Results

### Relation of individual lifestyle and weight control behaviors to BMI, weight loss, diet, and physical activity

Analyses were done with lifestyle and weight control behaviors treated as continuous variables and as dichotomous high and low categories taking into account the response scales and reported frequencies of each behavior. The strength and direction of associations were very similar in the two analyses. We present the categorical results here because we felt that differences between categorical means would be easier to interpret than beta weights. For high frequency behaviors such as eating meals, the variables were dichotomized into *< daily* and *daily*. For low frequency behaviors such as eating food from a convenience store, the variables were dichotomized into *none* and *1 +* times per week. For medium frequency behaviors such as eating after 7:00 p.m., the variables were dichotomized into *< 3* and *3 +* times per week. Table 2 shows the estimated means of BMI, weight loss, diet, and physical activity as a function of individual lifestyle and weight control behavior variables. As shown in Table 2, most behaviors were related to at least one outcome of interest and many were related to multiple outcomes. The behaviors most strongly related to BMI were watching television [< 2 hours/day *M* = 27.9 kg/m^2^ versus 2 or more hours/day *M* = 29.2 kg/m^2^, *F* (1,410) = 7.46, *p* < .01, *d* = .26] and eating food from a convenience store [none *M* = 28.2 kg/m^2^ versus 1 or more/week *M* = 30.1 kg/m^2^, *F* (1,411) = 8.11, *p* < .01, *d* = .39]. The behaviors most strongly related to percentage of weight lost in the last year were eating fast food [none *M* = 17.0% versus 1 or more/week *M* = 15.3%, *F* (1,412) = 10.17, *p* < .01, *d* = .32] and the weight control strategies of writing down calorie content, [rarely or less *M* = 15.7% versus sometimes or more *M* = 17.6%, *F* (1,412) = 10.17, *p* < .01, *d* = .37], using meal replacements [rarely or less *M* = 15.8% versus sometimes or more *M* = 17.4%, *F* (1,412) = 6.02, *p* < .05, *d* = .30], and planning meals [sometimes or less *M* = 15.0% versus often or more *M* = 16.8%, *F* (1,412) = 9.48, *p* < .01, *d* = .33]. The behaviors most strongly related to fat and sugar intake were eating food from a convenience store [none *M* = -0.07 z-score versus 1 or more/week *M* = 0.42 z-score, *F* (1,412) = 14.06, *p* < .001, *d* = .49] or from a fundraiser [none *M* = -0.03 z-score versus 1 or more/week *M* = 0.55 z-score, *F* (1,412) = 7.46, *p* < .01, *d* = .58] and planning meals [sometimes or less *M* = 0.20 z-score versus often or more *M* = -0.10 z-score, *F* (1,412) = 8.72, *p* < .01, *d* = .30]. The behaviors most strongly related to fruit and vegetable intake were eating lunch [less than daily *M* = -0.21 z-score versus daily *M* = 0.13 z-score, *F* (1,412) = 11.53, *p* = .001, *d* = .34], eating fast food [none *M* = 0.17 z-score versus 1 or more/week *M* = -0.17 z-score, *F* (1,412) = 11.42, *p* = .001, *d* = .34], and the weight control strategies of writing down calorie content [rarely or less *M* = -0.09 z-score versus sometimes or more *M* = 0.28 z-score, *F* (1,412) = 10.66, *p* = .001, *d* = .37] and planning meals [sometimes or less *M* = -0.26 z-score versus often or more *M* = 0.14 z-score, *F* (1,412) = 14.68, *p* < .001, *d* = .40]. Finally, the behaviors most strongly related to physical activity were the weight control strategies of writing down exercise [rarely or less *M* = 1680 kcal versus sometimes or more *M* = 2486 kcal, *F* (1,412) = 23.02, *p* < .001, *d* = .48] and planning exercise [sometimes or less *M* = 1236 kcal versus often or more *M* = 2459 kcal, *F* (1,412) = 58.46, *p* < .001, *d* = .72].

**Table 2:** BMI, % recent weight lost, diet, and physical activity means as a function of lifestyle and weight control behaviors

**Lifestyle behaviors**	**Level**	**BMI**		**% weight lost**		**Fat & sugar (z-scores)**		**Fruits & veggies (z-scores)**		**Physical activity (kcal per week)**	
Eat breakfast	< daily	29.0		15.6†		0.02		−0.15*		1743*	
	daily	28.2		16.5		−0.01		0.10		2156	
Eat lunch	< daily	29.0†		15.6†		0.01		−0.21***		1938	
	daily	28.1		16.5		−0.01		0.13		2054	
Eat dinner	< daily	29.1		16.0		−0.01		−0.16*		2017	
	daily	28.3		16.2		0.01		0.07		1993	
Eat after 7 p.m.	< 3/week	28.0†		15.6†		−0.03		−0.001		1934	
	3 +/week	28.9		16.6		0.02		0.01		2043	
Snack with TV	< 3/week	28.4		15.8†		−0.06†		−0.04		2095	
	3 +/week	28.7		16.8		0.11		0.09		1847	
Meal with TV	< 3/week	28.2		16.3		−0.04		−0.07		2020	
	3 +/week	28.7		16.1		0.04		0.09		1987	
TV viewing	< 2 hr/day	27.9**		16.6†		−0.07†		0.06		2150*	
	2 + hr/day	29.2		15.6		0.09		−0.06		1826	
Food from work	none	28.6		16.2		0.01		0.08†		1996	
	1 +/week	28.3		16.1		−0.01		−0.10		2005	
Food from sit down	none	28.4		16.1		−0.06		0.01		1883	
	1 +/week	28.5		16.2		0.03		0.004		2026	
Food from fast food	none	28.1†		17.0**		−0.07		0.17***		2171*	
	1 +/week	28.9		15.3		0.08		−0.17		1818	
Food from convenience store	none	28.2**		16.3		−0.07***		0.02		2017	
	1 +/week	30.1		15.5		0.42		−0.10		1904	
Food from fundraiser	none	28.6		16.2		−0.03**		0.01		2020	
	1 +/week	27.4		15.4		0.55		−0.10		1621	
Self-weighing	< daily	28.7		16.3		0.09†		0.001		1900	
	daily or more	28.3		16.0		−0.09		0.01		2110	
Write down calorie content	rarely or less	28.5		15.7***		0.05†		−0.09***		1893*	
	sometimes or more	28.3		17.6		−0.14		0.28		2315	
Write down exercise	rarely or less	28.4		16.0		0.03		−0.03		1680***	
	sometimes or more	28.6		16.4		−0.04		0.06		2486	
Use meal replacements	rarely or less	28.4		15.8*		0.001		−0.002		2044	
	sometimes or more	28.9		17.4		0.01		0.02		1834	
Plan meals	sometimes or less	29.2*		15.0**		0.20**		−0.26***		1720*	
	often or more	28.1		16.8		−0.10		0.14		2142	
Plan exercise	sometimes or less	28.8		15.5*		0.11†		−0.08		1236***	
	often or more	28.3		16.6		−0.06		0.05		2459	

All ANOVAs control for age, gender, race (white versus not), and marital status (married versus not). Significance level of mean differences: † *p* < .10, * *p* < .05, ** *p* < .01, *** *p* < .001.

### Factor analysis of lifestyle and weight control variables

Although the mean comparisons reported in Table 2 provide some insight into individual behaviors associated with BMI, recent weight loss, diet, and physical activity, the multiple comparisons and imperfect behavioral indicators make overall interpretation difficult. Therefore, factor analysis was used to gain a clearer understanding of how the behaviors in question relate to one another and to identify general lifestyle patterns. A series of analyses using principal component analysis and promax rotation (allowing the factors to be correlated) were performed to determine the simple structure underlying the observed correlations of lifestyle and weight control behaviors (i.e., to reduce the number of variables to a smaller number of factors which account for a large amount of the variability). Based on a scree plot (there was a clear break between factors four and five) and pattern of salients (i.e., the pattern of loadings with various numbers of factors specified), a 4-factor solution seemed to best account for the observed correlations (see Table 3 for factor loadings). The four factors accounted for 43% of the item variability and we labeled them as 1) regularity of meals, 2) television related eating and viewing, 3) intentional weight control strategies, and 4) eating away from home. Only one behavior, self-weighing, did not have a factor loading of at least .30 on any factor (it loaded .26 on the weight control strategies factor). The regularity of meals factor was positively related to the weight control strategies factor (*r =* .25, *p* < .001) and negatively related to the eating away from home factor (*r =* -.26, *p* < .001). The weight control strategies factor was negatively related to the eating away from home factor (*r =* -.14, *p* < .01). The television related eating and viewing factor and the eating away from home factor were positively related (*r =* .20, *p* < .001).

**Table 3:** Factor loadings for lifestyle and weight control behaviors

**Item**	**Regularity of meals**	**TV related viewing & eating**	**Weight control strategies**	**Eating away from home**
Eat lunch	.87			
Eat dinner	.81			
Eat breakfast	.70			
Eat a snack while watching TV		.78		
Eat a meal while watching TV		.76		
Average daily hours of TV viewing		.75		
Eat after 7 p.m.		.52		
Write down amount and type of exercise			.76	
Write down calorie content of food			.70	
Plan meals to manage weight			.48	
Plan exercise to manage weight			.47	−.35
Use meal replacements to manage weight			.41	
Self-weighing				
Eat food prepared at sit down restaurant				.64
Eat food at work (provided by employer or other employee)				.60
Purchase food at convenience store/gas station				.50
Eat food prepared at fast food restaurant				.44
Purchase food for a fundraiser				.32

Only loadings above .3 are shown.

### Relation of factors to BMI, weight loss, diet, and physical activity

Having established that the lifestyle and weight control behaviors formed coherent, meaningful factors, we examined the relation of these factors to outcomes of interest (controlling for age, gender, race, and marital status). Similar to the analyses examining individual behavioral predictors, the lifestyle factors were treated both continuously (factor scores were computed from the analysis described above using the regression method, which predicts the location of each individual on each factor) and categorically (we divided each of the factor scores into tertiles, i.e., 3 equal groups), and the strength and direction of effects were quite similar in both cases. We again present the categorical results here because we felt that differences between categorical means would be easier to interpret than beta weights.

Table 4 shows the means of BMI, recent weight loss, diet, and physical activity broken down by tertiles of the lifestyle factor scores for regularity of meals, television related eating and viewing, weight control strategies, and eating away from home. Eating more regular meals was related to greater weight loss in the last year [lower *M* = 15.9% and middle *M* = 15.4% versus upper *M* = 17.3%, F(2,402) = 4.72, *p* < .05, *ds* = .26, .34] and greater fruit and vegetable intake [lower *M* = -0.22 z-score versus middle *M* = 0.13 z-score and upper *M* = 0.13 z-score, F(2,402) = 5.59, *p* < .01, *d’s* = .35, .35]. More use of weight control strategies was associated with a lower BMI [lower *M* = 29.2 kg/m^2^ versus middle *M* = 27.6 kg/m^2^, F(2,401) = 3.61, *p* < .01, *d* = .32], greater weight loss in the last year [lower *M* = 14.9% versus middle *M* = 16.7% and upper *M* = 17.0%, F(2,402) = 6.63, *p* = .001, *ds* = .35, .41], lower fat and sugar intake [lower *M* = 0.20 z-score versus middle *M* = -0.11 z-score and upper *M* = -0.08 z-score, F(2,402) = 4.47, *p* < .05, *ds* = .31, .28], greater fruit and vegetable intake [lower *M* = -0.18 z-score versus upper *M* = 0.20 z-score, F(2,402) = 5.07, *p* < .01, *d* = .38], and greater physical activity [lower *M* = 1411 kcal versus middle *M* = 1964 kcal versus upper *M* = 2612, F(2,402) = 18.87, *p* < .001, *ds* = .33 (lower versus middle), .38 (middle versus upper), .71 (lower versus upper)]. Conversely, greater television related viewing and eating was related to greater BMI [lower *M* = 27.8 kg/m^2^ and middle *M* = 28.0 kg/m^2^ versus upper *M* = 29.5, F(2,401) = 5.11, *p* < .01, *ds* = .35, .31] and higher fat and sugar intake [lower *M* = -0.22 z-score versus middle *M* = 0.09 z-score and upper *M* = 0.14 z-score, F(2,402) = 5.90, *p* < .01, *ds* = .31, .36]. More eating away from home was related to greater fat and sugar intake [lower *M* = -0.16 z-score versus upper *M* = 0.19 z-score, F(2,402) = 4.77, *p* < .01, *d* = .35], lower fruit and vegetable intake [lower *M* = 0.11 z-score and middle *M* = 0.08 z-score versus upper *M* = -0.16 z-score, F(2,402) = 3.07, *p* < .05, *ds* = .27, .24], and less physical activity [lower *M* = 2294 kcal and middle *M* = 2106 kcal versus upper *M* = 1586 kcal, F(2,402) = 6.70, *p* = .001, *ds* = .42, .31].

**Table 4:** BMI, % recent weight lost, diet, and physical activity means as a function of lifestyle and weight control factors

**Factor**	**Tertile**	**BMI**		**% weight lost**		**Fat & sugar (z-scores)**		**Fruits & veggies (z-scores)**		**Physical activity (kcal per week)**	
Regularity of meals	Lower	29.0		15.9_a_		0.04		−0.22_a_		1962	
	Middle	28.6†		15.5_a_*		0.06		0.13_b_**		1777†	
	Upper	27.7		17.3_b_		−0.09		0.13_b_		2253	
TV related eating and viewing	Lower	27.8_a_		16.4		−0.22_a_		−0.05		2168	
	Middle	28.0_a_**		16.0		0.09_b_**		0.04		2011	
	Upper	29.5_b_		16.3		0.14_b_		0.04		1813	
Weight control strategies	Lower	29.2_a_		14.9_a_		0.20_a_		−0.18_a_		1411_a_	
	Middle	27.6_b_*		16.7_b_***		−0.11_b_*		0.02_a,b_**		1964_b_***	
	Upper	28.5_a,b_		17.0_b_		−0.08_b_		0.20_b_		2612_c_	
Eating away from home	Lower	27.8		16.7		−0.16_a_		0.11_a_		2294_a_	
	Middle	28.7		16.4		−0.02_a,b_**		0.08_a_*		2106_a_***	
	Upper	28.8		15.6		0.19_b_		−0.16_b_		1586_b_	

All ANOVAs control for age, gender, race (white versus not), and marital status (married versus not). Significance level for overall ANOVAs: † < .10, * *p* < .05, ** *p* < .01, *** *p* < .001. Means with different subscripts differ at *p <* .05.

## Discussion

The analyses presented in this paper used factor analysis to combine variables that have previously been found to be associated with body weight and risk of weight gain [9-19]. Twelve questions about regularity and location of eating as well as TV viewing and six questions about intentional strategies used for weight control were factor analyzed. A four-factor solution seemed to best fit the data, which we labeled regularity of meals, TV viewing and eating, intentional strategies for weight control, and eating away from home. These factors were examined in relation to degree of success in a recent weight control attempt, food intake, physical activity, and current BMI. These analyses clearly showed that variables with heuristic similarity tend to group together, e.g., individuals who eat breakfast regularly also tend to eat other meals more regularly. Qualitatively, it appears that combining these variables into meaningful groupings produced variables that were related to behavioral and weight outcomes more consistently than the individual variables from which they were derived. For example, the weight control factor was consistently related to all weight, diet, and physical activity variables, whereas the individual weight control behaviors varied substantially in terms of significant associations with weight, diet, and physical activity variables. Additionally, whereas none of the individual meals variables were significantly related to recent weight loss, the regularity of meals factor was significantly related to recent weight loss.

Most of the observed associations with outcomes were in the direction that would be expected, as were their associations with each other. There were a few surprises, however. For example, TV viewing and eating was strongly associated with BMI and fat and sugar intake, but not with success in recent weight control efforts or fruit and vegetable intake and physical activity. Eating regular meals was associated with greater success in recent weight control efforts and higher fruit and vegetable intake, but was not associated with fat and sugar intake and only weakly related to BMI and physical activity. Greater eating away from home was associated with less healthful eating and physical activity behaviors, but was not significantly related to BMI or recent weight loss (although the trends are in the expected direction). Intentional weight control strategies were most consistently related to healthier behaviors, healthier BMI, and greater success in recent weight control efforts. The latter finding is clearly supportive of intentional weight control efforts as healthy behavior. However, all participants in this study were required to have lost 10% or more of their body weight within the last year, which excluded any individuals who tried to lose weight but without any success.

Including only successful losers in this sample poses clear problems with generalizing from these results to the general population. The sample is also fairly high in SES and weighted toward women. While the sample has limitations, we believe it also offers a unique insight into the processes of self-initiated weight loss and suggests that even among those who try to lose weight and are successful there is still a dose response relationship between degree of weight control effort and recent weight loss and BMI. Unexpectedly, self-weighing did not load highly onto the weight control factor and was not related to weight in this sample. However, there may have been a restriction of range issue for this behavior as most participants engaged in at least weekly weighing (88%), and the other weight control behaviors had greater variability. In future research it will be important to examine this diverse array of lifestyle behaviors in a more representative sample.

The results of this study do not answer precisely the question about obesity risk phenotypes. However, they suggest to us some possible directions for additional research. For example, regularity of eating meals was related to greater weight loss in the past year, whereas eating out was related to poorer diet and exercise habits and TV related eating and viewing was related to greater BMI. This suggests that individuals with more ordered eating lives may have more weight control success. Is having an ordered eating life an indication of a broader mastery of self-control skills, or a better sense of proportion in making commitments? It also appears that meal regularity and weight control strategies were relatively more strongly associated with the positive outcomes of recent weight loss and eating fruits and vegetables, whereas TV related eating and viewing and eating away from home were relatively more associated with the negative outcomes of BMI and fat and sugar intake. This pattern of results suggests that the identified behavioral factors can be thought of in terms of relatively more regulated and unregulated lifestyle patterns (correlation among the factors also suggests this). Thus, an important question is the extent to which these patterns also differentially predict weight control over time? Other research suggests that disordered lifestyle patterns (e.g., disordered work schedule, disordered meal patterns) are related to weight gain over time [16,19,32].

We have used factor analysis, specifically, principal component analysis, in an attempt to simplify and organize a diverse set of behaviors. Other data reduction techniques could have been used to examine the data. For example, one might wish to identify behavioral clusters based on a particular outcome of interest. In this way, one could identify groups of behaviors that discriminate between eating fruits and vegetables or not or exercising a lot or a little. One might also wish to try to cluster individuals together based on a number of different behaviors. In this way one could examine diet, physical activity, and weight for particular clusters of people. For example, Sanchez et al. [22] found that overweight and obese women were likely to have multiple lifestyle risk behaviors (e.g., not meeting dietary and physical activity guidelines) and Boone-Heinonen et al. [24] found that prevalent and incident obesity were related to behavioral clusters (based on a number of diet and physical activity behaviors) in adolescent boys and girls. Given the cross-sectional nature of our data and our focus on a diverse set of lifestyle behaviors, factor analysis was an appropriate method for identifying unifying themes.

Recognizing that obesity is a multifaceted problem, it may also be important to link these behavioral patterns to environmental (e.g., availability of convenience foods, structural features of neighborhoods) and psychological influences (e.g., disinhibition, restraint, susceptibility to hedonically pleasing foods) that have been implicated in obesity risk [4,5,33-35]. In this way, the pattern of influences on obesity can be more fully established (e.g., environmental and psychological/biological influences → behavioral patterns → diet and physical activity → weight, weight change). For example, higher levels of disinhibition with respect to food are associated with current and prospective weight gain [33,34]. It may be that our TV and eating away from home factors are associated with psychological disinhibition and lead to weight gain over time.

Drawing on distinctions between behavioral initiation and maintenance [18,36], we aim to establish the relation of these behavioral patterns to weight change over time. Furthermore, we plan to examine factor change over time and the relation of factor change to weight maintenance. For example, TV related eating and viewing was related to BMI, but was unrelated to the current weight loss episode. Over time will this group of behaviors predict weight gain? Furthermore, will these factors have an overall effect on weight gain? Research using large cohorts suggests that various lifestyle behaviors (e.g., specific dietary components, physical activity, and sleep) have a substantial aggregate effect on weight gain over time [37]. It is important to note that research examining aggregate effects of behavior on weight over time [37] or the differential effects of particular behaviors with weight loss and weight maintenance [18] have not examined the effects of behavioral factors (derived via factor analysis) on weight over time.

## Conclusions

In a study of people who lost substantial weight of their own initiative (at least 10 percent in the past year), we were able to identify four lifestyle and weight control behavioral factors related to obesity—regularity of meals, weight control strategies, TV related eating and viewing, and eating away from home. Compared to the individual behavior variables, these factors appeared to be more reliably related to BMI, recent weight loss, diet, and physical activity. In general, eating away from home and TV related eating and viewing were more related to negative outcomes (BMI, fat and sugar intake), whereas eating regular meals and using weight control strategies were more related to positive outcomes (current weight loss, eating fruits and vegetables). Greater use of weight control strategies was most consistently related to better weight, diet, and physical activity outcomes. These findings add to the growing body of literature identifying behavioral patterns related to obesity risk and the overall strategy of eating less and exercising more [18,37]. In future research it will be important to replicate these behavioral factors (over time and in other samples) and to examine how changes in these factors relate to weight loss and weight maintenance over time.

## Competing interests

The authors declare they have no competing interests.

## Authors’ contributions

NES and RWJ contributed to the conception and design of the project. PTF performed the analysis and all authors contributed to the interpretation. PTF and RJW drafted the manuscript and NES provided critical revision. All authors edited and approved the final manuscript.
